# *Tropilaelaps mercedesae* Infestation Is Correlated with Injury Numbers on the Brood and the Population Size of Honey Bee *Apis mellifera*

**DOI:** 10.3390/ani13081318

**Published:** 2023-04-12

**Authors:** Tial C. Ling, Patcharin Phokasem, Chainarong Sinpoo, Panuwan Chantawannakul, Kitiphong Khongphinitbunjong, Terd Disayathanoowat

**Affiliations:** 1 Bee Protection Laboratory, Department of Biology, Faculty of Science, Chiang Mai University, Chiang Mai 50200, Thailand; 2 School of Science, Mae Fah Luang University, Chiang Rai 57100, Thailand

**Keywords:** *Apis mellifera*, bee population, crippled honey bee, infestation, injury numbers, larvae, pupae, *Tropilaelaps mercedesae*

## Abstract

**Simple Summary:**

A global decline in the population of bee pollinators is regarded as a potential threat to species extinction and global food security. Mite infestation plays a vital role in contributing to the collapse of bee populations. However, the correlation between bee population and mite infestation remains unclear. This study investigated *Tropilaelaps mercedesae* mite infestations to the larval, pupal, and crippled adult stages of honey bee *Apis mellifera*, the relationship between mite infestation rate and injury numbers for each of bee larvae and pupae, and the relationship between mite infestation rate and population size per beehive. Mite infestations occurred in all developmental stages of the honey bees, and it was pronounced in the abdomens and the antennas of the honey bees. Mite infestation rate was positively correlated with the number of injuries per bee in each of the larvae and pupae and negatively correlated with the population size per beehive. Overall, our findings suggested that the use of a large population size of beehives can reduce the infestation rate. It also provided important information about the adaptation of mite/antibacterial immune competence of honey bees to different life stages and the breeding stock of bees for hygienic behaviors resisting mite infestations.

**Abstract:**

*Tropilaelaps mercedesae*, one of the most devastating parasitic mites of honey bee *Apis mellifera* hosts, is a major threat to honey products by causing severe damage to honey bee colonies. Here, we recorded injury numbers caused by *T. mercedesae* to different body parts of the larval, pupal, and crippled adult stages of honey bee *A*. *mellifera*. We evaluated the relationship between infestation rate and injury numbers per bee for both larvae and pupae. We also noted the total bee numbers per beehive and examined the relationship between the infestation rate and population size. *T*. *mercedesae* infested all developmental stages of honey bees, with the highest injury numbers in the abdomens of bee pupae and the antennas of crippled adult bees. Although larvae received more injury numbers than pupae, both infestation rate and injury numbers decreased as the larval stage progressed to the pupal stage. The infestation rate increased as the population size per beehive decreased. This study provided new perspectives to the understanding of changes in the effects of *T*. *mercedesae* infestations on different developmental stages of honey bees. It also showed useful baseline information for screening honey bee stock that might have high defensive behaviors against mite infestation.

## 1. Introduction

Bee pollinators, especially *Apis* species, are the key players in the crop yield process and important vectors in maintaining the natural balance of ecosystems. A consistent decline in the populations of honey bees (also known as *Apis* species) and other bee species has been demonstrated worldwide, causing a potential threat of species extinction and a risk to global food security [1,2,3,4]. Parasite infestation is one of the most critical factors that have led to bee population decline worldwide [5,6]. Previous studies have postulated that parasitic mites in the genus *Tropilaelaps* and *Varroa* are the main factors that severely damage honey bee colonies in the continent of Asia [7,8,9,10,11,12,13].

*Tropilaelaps*, a genus of parasitic mites in the family Laelapidae, is often regarded as a major threat to honey products by causing severe damage to honey bee colonies in Asia [11,14,15,16,17]. *Tropilaelaps* (e.g., *T*. *mercedesae*) mites originally parasitized the brood of the Asian giant honey bee *Apis dorsata* [9,18]. These mites have transferred to the European honey bee *A*. *mellifera* [9,19] and have been found to infest both temperate and tropical populations of *A*. *mellifera* in Asia [8,9,10,20]. In general, the life cycle and reproductive strategies of *T*. *mercedesae* are similar to that of *Varroa* mites. They both feed on the brood of honey bees and are vectors for the Deformed Wing Virus (DWV) [9,21]. However, unlike *Varroa* mites, *T*. *mercedesae* mites have smaller size, more rapid locomotion, and a greater reproductive rate [9,20,22,23], and because of these, their population growth can be even faster than those of *Varroa* mites [20,24]. Moreover, the life span of *T*. *mercedesae* mite is shorter than that of *Varroa* [16,25,26]. Thus, *T*. *mercedesae* mites spend most of their life in the capped brood cells of their host and infest honey bees by sucking out the hemolymph of developing honey bees in the colony [9,27], and thus they consume the body resources of bees. Previous studies suggested that the major impact of *Tropilaelaps* mite infestation is caused by the mite itself, reducing bee host immune responses (e.g., [28]). Honey bee’s hemolymph is composed of several nutrients (e.g., carbohydrates and proteins) and different hemocyte types which are crucial for the immunity of honey bees [29,30,31,32]. However, *A*. *mellifera* lacks behavioral mechanisms to defend against *T*. *mercedesae* mite infestations [16]. For this reason, like *Varroa* destructor, *T*. *mercedesae* mite infestations often cause both abnormal brood development and brood death in honey bee *A*. *mellifera* such as deformed pupae and adults (e.g., stunting, damaged abdomens/antennas/legs/wings), parasitic mite syndrome, and colony health [8,19,21,27,33]. These effects are regarded to be developed through mites feeding on the hemolymph of the bee and also by spreading honey bee viruses, particularly DWV [34,35]. Mite infestations have also been found to reduce protein concentrations, longevity, and weight in the pupal stage of honey bees [8,27,36]. A previous study suggested that either a decrease in carbohydrate and fat contents or an increase in protein content and defense mechanisms of the immune systems of *A*. *mellifera* was accompanied by honey bees at different developmental stages from the larval to adult stages [37]. Therefore, mite infestation is likely to vary across different life stages of the bees depending on their nutrient compositions and defense mechanisms of immune systems against the infestation. If nutrient compositions and defense mechanisms or hygienic behaviors against mite infestations are weak in the early developmental stage of the honey bees, the infestation rate (the proportion of infested bees per hive) caused by *T*. *mercedesae* should be higher in bee larvae than in bee pupae and adult bees. However, the potential for variations in infestations caused by *T*. *mercedesae* in the different developmental stages of the honey bees is rarely studied.

It has been postulated that honey bees infested by *T*. *mercedesae* during the early developmental stage enhance viral proliferation in the beehive through long exposure to the virus and the stress on susceptibility to viral infection rates [38]. Thus, variations in mite infestation rates may influence the number of injuries of honey bees across different developmental stages, which would lead to the spreading of the viruses and further affect the colony’s health and honey products. Moreover, injury numbers may vary among different body parts of the bees, as each body part is structured with different organs and functions differently [8]. A previous study on Thailand populations of *A*. *mellifera* suggested that the number of injuries was positively correlated with the number of actively feeding mites in both the fifth instar larvae and pupae of the bees. Among different body parts of the bees, the highest injury numbers were found in the abdomens and the antennas, whereas the lowest injury numbers were found in the thoraxes and the mouth parts [8]. However, it remains unknown whether the *T*. *mercedesae* infestation rate is correlated with the injury numbers on the brood and the population size (the total number of bees per hive) of *A*. *mellifera* bees. It is postulated that abiotic factors (e.g., temperature and humidity) have a great impact on the population level, survivorship, and fecundity of mites, such that high temperatures in the colonies could reduce mite growth [39,40]. A previous study also noted that bees with large colonies increased the temperature level of the colonies when compared to those with small colonies [41]. In addition, the hygienic behaviors of the bees have been found to influence mite infestations [40]. Therefore, bees with small population sizes, relative to those with large population sizes, are expected to have a higher infestation rate and injury numbers in the body parts of the individual bees due to the potential for the reduction in relative temperature and humidity, and hygienic characteristics of the colonies. If bee pupae had the highest number of injuries in the abdomens and antennas [8], the greatest number of injuries should also occur in the abdomens and antennas of adult bees and the lowest in the thoraxes and mouth parts, as they both have almost identical morphological features, however, this remains unclear.

In the present study, we documented the number of injuries (both fresh wounds and scars) caused by *T*. *mercedesae* to the bee larvae (i.e., fifth instar larvae and prepupae) and different body parts (i.e., abdomens, antennas, thoraxes, mouth parts, legs, and wings) of the pupal and crippled adult stages of *A*. *mellifera*. We also examined the *T*. *mercedesae* mite infestation rate, the correlation between mite infestation rate and the number of injuries per bee for both larvae and pupae and the relationship between mite infestation rate and bee population size. We hypothesized the highest injury numbers in the abdomens and the antennas of the bees, the lowest injury numbers in the thoraxes and mouth parts of the bees, a positive correlation between mite infestation rate and injury numbers for both bee larvae and pupae, and a positive relationship between the infestation rate of mites and the population size of honey bee *A*. *mellifera*.

## 2. Materials and Methods

### 2.1. Study Sites and Source of Honey Bee Samples

Samples of the larval and the pupal stages of honey bees (*Apis mellifera*) from hives were collected at Chan Chawa in the Chiang Rai province of Northern Thailand and that of newly emerged crippled adult bees from hives were collected in Chiang Mai city of Northern Thailand. Both field and laboratory observations of injury numbers of the bee larvae and pupae were performed between May and July 2017, while that of the adult honey bees was evaluated between February 2017 and March 2018. In this study, we used a queen-right Langstroth hive of a honey bee colony consisting of eight to ten movable frames. Each frame contained different developmental stages of bees such as 5th instar larvae, prepupae, white-eyed pupae, pink-eyed pupae, purple-eyed pupae, older pupae, and crippled adult bees. Fifth instar larvae and prepupae were defined as the larval stage of honey bees, whereas white-eyed pupae, pink-eyed pupae, purple-eyed pupae, and older pupae were defined as the pupal stage. We used fifteen randomly selected beehives for bee larvae and pupae as well as eight randomly selected beehives for adult bees to examine injury numbers. Before the study, all selected beehives were previously infested with mites. Injury numbers of larvae and pupae were examined at the laboratory of the School of Life Science at Mae Fah Luang University, while that of adult bees were explored at Bee Protection Laboratory at Chiang Mai University. All beehives and the observation of mite infestations at each developmental stage of bees were monitored on the same day or one close to the day to minimize variations in the number of mite infestations among bees or beehives.

### 2.2. Examination of Population Size, Injury Numbers, and Infestation Rate in Larvae and Pupae

We examined the population size of adult honey bees following the method developed by Delaplane et al. [42]. Briefly, we visually estimated the percentage of adult bees on both sides of a comb of all individual frames in each colony. All percentages were pooled as a total percentage, then the population size was calculated by multiplying the total percentage of adults by bee numbers per fully occupied side of the Langstroth.

To observe injuries caused by *Tropilaelaps* mites to the larvae and different body parts (i.e., abdomens, antennas, legs, mouth parts, thoraxes, and wings) of the pupae, we selected one frame from the middle Langstroth hive for each of the fifteen beehives. From each selected brood frame, we randomly collected 35 infested larvae and pupae and recorded their injury numbers and locations under a stereo microscope. In this study, fresh wounds (a fresh injury to the skin or body tissue of the honey bees) or scars (a mark left on the skin or within body tissue where a wound has not healed completely, and fibrous connective tissue has developed) caused by *T*. *mercedesae* mites to any parts of a bee’s body was defined as injury. Before the examination of injured bees, all infested larvae and pupae were extracted from brood cells with blunt-pointed dissection forceps (Union Science Ltd., Muang Chiang Mai 50200, Thailand), taking extreme care to avoid damage to any part of their body. All signs of wounding in developmental stages were considered as damaged/injured brood which then was removed by destroying the surrounding wax cell and lifting the brood with the forceps. Moreover, the wounds on the broods were confirmed by immersing the brood in 0.4% trypan blue solution (Invitrogen, Carlsbad, CA, USA) for 30 min at room temperature according to the method developed by Ganbar and Engels [43]. This chemical solution stains the damaged epidermal cells of the bees caused by *Tropilaelaps* mites, enabling us to detect injury locations and numbers of both larvae and pupae. Fresh integumental wounds appeared as blue spots, while wounds that had healed and appeared brown to black were referred to as scars (Figure S1). The examination of injuries was conducted under a stereo microscope. Then, the number of injuries (both fresh wounds and scars) was recorded according to the corresponding location.

We justified our initial determination of mite infestation rate by uncapping 300 randomly selected worker brood cells per colony. Regardless of the number of mites in the cell, it was considered as one infested cell. The infestation rate was then calculated by dividing the total number of infested cells by the total number of both infested and uninfested cells for each frame in each beehive. The brood cells were collected from two to three randomly selected frames per beehive. A total of 35 frames from 15 beehives were used for the determination of the infestation rate.

### 2.3. The Observation of Injuries on Crippled Adult Honey Bees

Symptomatic bees with wing deformities or with normal wings that were small and sluggish [44] were observed in each of eight randomly selected beehives before the examination of injury numbers caused by *T*. *mercedesae* mites to newly emerged honey bees. We randomly collected 243 newly emerged crippled/mite-infested adult bees from eight monitored beehives using blunt-tipped forceps, taking extra care to avoid damage to bee bodies. We then recorded injury numbers that occurred in different body parts of crippled adult bees under a stereo microscope (an illustration of injury types in different body parts of the honey bees is shown in Figure S2).

### 2.4. Scanning Electron Microscope (SEM)

Based on the observation of injuries at different developmental stages of honey bees, we randomly selected *Tropilaelaps*-infested pupae and crippled adults for scanning electron microscope experiments. The pupal and adult tissues (i.e., wings and antennas) of honey bees were fixed into 1.0 mM glutaraldehyde in cacodylate-buffered, pH 7.2 for two hours. The fixed samples were vacuumed for better penetration for 2 h and then rinsed in the buffer for 30 min. After stepwise dehydration in the ethanol, we applied the critical point for CO_2_. The dried pupal and adult tissues were mounted and sputtered with gold palladium. The photographs of injured tissues were taken using the scanning electron microscope (SEM: JSM-IT300) [45].

### 2.5. Data Analyses

All statistical analyses were performed in R version 4.1.2 (R Development Core Team, 2022), and data were expressed as the mean ± standard deviation. Independent sample *t*-tests were used to test for significant differences in injury numbers either between the larval and pupal stages of honey bees or between 5th instar larval and prepupal stages of the bees. A generalized linear model (GLM) with Poisson errors was used to test the differences in the number of injuries among body parts (abdomens, antennas, thoraxes, legs, mouth parts, and wings) and types of injury (fresh wounds and scars) for bee pupae. We divided neither body parts nor injury types when analyzing injury numbers of the bee larvae. The interaction between the body parts and injury types was also included. When significant differences were found, differences between levels of each effect were analyzed using multiple comparisons of means with Tukey contrasts using a “glht” function in the “multcomp” package [46]. We used GLM with Binomial errors to test for the relationship between the infestation rate and injury numbers for each of the larval and pupal stages as well as the relationship between infestation rate and population size. Kruskal–Wallis tests were employed to compare injury numbers among different body parts of the adult honey bees. We did not find any injuries on the thoraxes and the mouth parts of adult bees and only a single injury was detected on the legs from a single honey bee. We, therefore, excluded the data for thoraxes, mouth parts, and legs from statistical analysis to increase statistical power.

## 3. Results

### 3.1. Bee Population, Number of Injuries in Larvae, Pupae, and Crippled Adult Honey Bees

The population size of honey bee *Apis mellifera* ranged from 19,712 and 32,472 per hive. The mean number of injuries in fifth instar larvae (13.16 ± 8.61, *n* = 18) was much lower than that of injuries in prepupae (31.59 ± 21.20, *n* = 88) (t = −3.616, df = 104, *p* < 0.001). Injury numbers varied among body parts of the bee pupae (Wald chi-square = 339.740, df = 3, *p* < 0.001), with the highest number of injuries in abdomens (2.80 ± 3.96, *n* = 174), followed by antennas (0.46 ± 0.97, *n* = 174), legs (0.13 ± 0.84, *n* = 174), thoraxes (0.05 ± 0.45, *n* = 174), and mouth parts (0.03 ± 0.02, *n* = 92), respectively. The wings of the bee pupae had no injury. When the injury of the bee pupae was further divided into fresh wounds and scars, the overall mean number of fresh wounds (0.69 ± 2.47, *n* = 348) was significantly more than that of scars (0.13 ± 2.23, *n* = 348) (Wald chi-square = 30.395, df = 1, *p* < 0.001) although their interaction was significant (Wald chi-square = 39.785, df = 3, *p* < 0.001). Except for fresh wounds (2.89 ± 3.57, *n* = 87) and scars (2.71 ± 4.34, *n* = 87) in the abdomens of the pupae, the antennas, the thoraxes, and the legs of the pupae received significantly higher numbers of fresh wounds than that of scars (0.81 ± 3.57 for fresh wounds and 0.26 ± 0.72 for scars, *n* = 87 for antennas; 0.21 ± 0.61 for fresh wounds and 0.01 ± 0.11 for scars, *n* = 87 for thoraxes; and 0.48 ± 1.13 for fresh wounds and 0.04 ± 0.18 for scars, *n* = 87 for legs).

**Figure 1:**
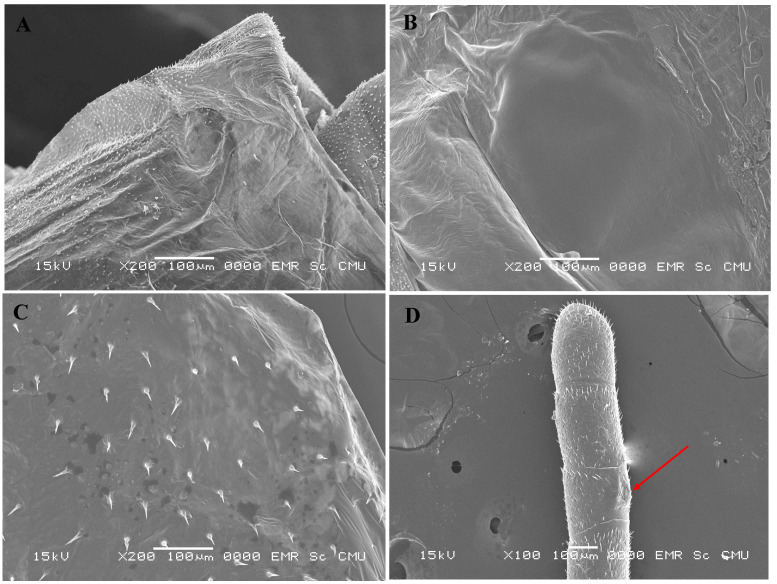
Scanning electron microscope pictures of *A*. *mellifera* tissues. (**A**) The abdomen of a purple-eyed pupa, (**B**) the abdomen of tanned-bodied pupa, (**C**) the wing of the crippled adult honey bee, and (**D**) the antenna of the crippled adult honey bee. The red arrow points to the scar of the antenna.

We found that 113 (46.50%) out of 243 crippled adult honey bees were injured. A significant difference was observed among the injury number of antennas, abdomens, and wings (Kruskal–Wallis: R2 = 958.66, df = 1, *p* < 0.001; Figure 1). The mean injury number in the antennas (0.61 ± 0.79, *n* = 243) was significantly more than that in the abdomens (0.07 ± 0.41, *n* = 243) and wings (0.07 ± 0.36, *n* = 243). There was no significant difference in the number of injuries between the abdomens and the wings of the bees.

### 3.2. The Relationship between Infestation Rate and Injury Numbers in Larvae and Pupae

**Figure 2:**
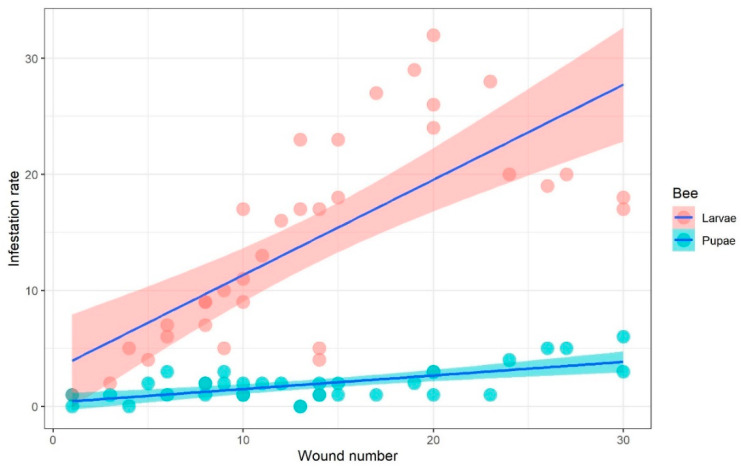
The relationship between the infestation rate of *Tropilaelaps mercedesae* mites and the number of injuries that occurred in each of the larval and pupal stages of honey bee *Apis mellifera*. Pink and cyan circles represent the infestation rate for each of the injury numbers of bee larvae and pupae. Blue solid lines with pink and cyan curves show the significant relationship between mite infestation rate and injury numbers of bee larvae and pupae using linear regression.

A significant relationship between mite infestation rate and injury numbers was observed for both bee larvae and pupae (Table 1). The infestation rate increased as the number of injuries increased for both larvae and pupae (Figure 2). The overall mean infestation rate was 14.26 ± 8.87 (*n* = 35) for larvae and 1.91 ± 1.44 (*n* = 35) for pupae. The infestation rate was significantly higher in larvae than in pupae (t = 8.126, df = 68, *p* < 0.001).

### 3.3. The Relationship between Infestation Rate and Bee Population Per Hive

**Figure 3:**
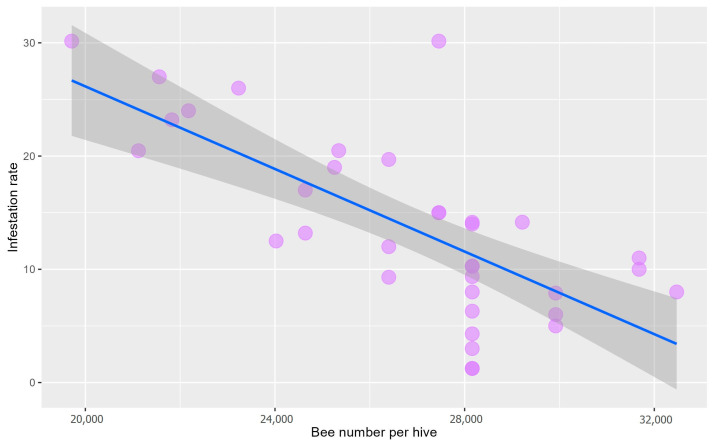
The relationship between *Tropilaelaps mercedesae* mite infestation rate and honey bee population (honey bee number) per beehive. Purple circles represent the infestation rate for each bee population size. Blue solid lines with gray loess curves show the significant relationship between mite infestation rate and bee population using linear regression.

There was a negative relationship between the population size of honey bee *Apis mellifera* and the infestation rate of *Tropilaelaps mercedesae* mites (Table 2). The infestation rate decreased as the number of honey bee individuals per colony increased (Figure 3).

## 4. Discussion

*Tropilaelaps* mites are widespread in honey *bee Apis mellifera* populations across many parts of Asia [20,27], and their infestations persist even in regions where winter can be harsh and brood production is limited [15,47]. These mites can have a high ability to reproduce in infested colonies [20,23]. In this study, unlike *Varroa* mites which only begin feeding after the sealed larvae consumed their larval food [48], but similar to a previous study [8], we found *T*. *mercedesae* mite infestations in all life stages of honey bees. Of these, the number of injuries to prepupae, also known as mature larvae, was significantly more than that to fifth instar larvae. This result was inconsistent with our hypothesis, where we expected a decreased number of wounds as the larvae progress to the adult stage. We postulated that increased injury numbers in the prepupal stage of honey bees could be related to feeding strategies on nutrients. For instance, the bee prepupae may have been exposed for a longer period for feeding on nutrient sources, in situations where food sources in the capped brood cells are limited. Another possible scenario is that poor hygienic behaviors [49,50,51], a mechanism of mite and disease resistance, in the pupal stage of honey bees might have caused a great number of injuries (Kitiphong et al. [10] and references therein). Consequently, the mites could infest the pupae when workers uncapped the brood cells, thereby affecting the cycle of feeding strategies.

Mite infestations in the capped brood cells of honey bee workers might also influence the emergence development and damage adult foraging ability such as crippled wings [21,27,44]. However, consistent with our prediction, we found a higher number of injuries in the bee larvae than in the bee pupae. Patcharin et al. [8] suggested that most injuries in mature larvae were healed when they molt during the white-eye pupal stage. In this study, we included both fresh wounds and scars when counting the number of injuries in the pupae, however, we still obtained a much lower number of wounds in the pupae compared to the number of wounds in the larvae. This result implies indirect support for the idea that bees might have developed mite or disease resistance as they progressed to the pupal stage. Nevertheless, we could confirm the reduction in the number of injuries accompanied by the development of honey bees from the larvae stage to the pupae stage of *A*. *mellifera*.

An increase in mite infestation rates can be expected where the presence of injuries on honey bees caused by parasitic mites is relatively high because these mites have rapid locomotion and reproductive rates [12,23]. Indeed, a previous study has reported that increased numbers of injuries are positively correlated with increased numbers of actively feeding mites [8]. Consistent with this trend, we found that the number of injuries in the bee larvae and pupae was positively correlated with the infestation rate of *T*. *mercedesae.* The more mites present, the higher number of injuries occurred in the bees. Specifically, the infestation rate was pronounced in the larvae, suggesting that changes in injury numbers could influence mite infestation rates at the larval and pupal stages of honey bees. In contrast, the infestation rate decreased as the honey bee population increased, showing the importance of bee colonies in response to mite infestations. However, it is important to remember that the infestation rate at the population level was evaluated during one specific time in this study. The present result might have been different if we based our estimation of brood infestations over a longer time because changes in the infestation rate might be effectively influenced by temporal variation in various mechanisms such as the density and hygienic behaviors of honey bees and the development of other unknown parasitic mechanisms (e.g., DWV) infesting mites/bees in the honey colonies. A previous study has suggested that bees with large colonies, relative to those with small colonies, have a greater density and movement (i.e., walking, resting, nursing, hive maintenance, worker maintenance, in festoon, and foraging) of workers and the ability to increase the temperature in the colonies [41]. Other studies have also reported that the development and population densities of mites are highly sensitive to weather conditions [39,40]. For example, the temperature was found to be crucial for the pest population levels in means of mite growth, survivorship, and fecundity of mites [39], while the hives’ location, humidity, and the beeline’s hygienic characteristics were also important for mite falls [40]. These patterns imply the fact that variations in mite infestation rates among bee colonies with different population sizes might be related to changes in the density and movement of workers, hygienic characteristics of the bees, and the temperature in bee colonies, which might subsequently influence colony defense mechanisms against mite/virus infestations. However, empirical investigations are needed to validate these hypotheses.

Although most injuries that occurred in mature larvae might have healed when they progressed to either the pupae stage or the adult stage, the combined effect of pre-capped and post-capped brood injuries might have damaged various body parts of the older pupal and adult stages of honey bees including wings, mouth parts, antenna, thoraxes, abdomens, and legs. In this study, we found a great number of injuries in the abdomens of the pupae. This result supports the previous finding [8] that injury numbers in the abdomens of the bee pupae of *A*. *mellifera* were significantly higher than that in other body parts of the honey bees. Injuries caused by *Tropilaelaps* mites to the pupal stage of the honey bees resulted in permanent injuries at the adult stage of the crippled honey bees, and it was commonly found in the bees’ antennas, wings, and abdomens. Of these, we found that the highest number of injuries was found in the antennas. Antennas are important paired sensory organs of workers and drones of adult honey bees, particularly responsive to stimuli, touch, and odor [52]. Therefore, antennal segment deformation may be affected by the behavioral strategies of honey bees. *Tropilaelaps* mite infestations showed a negative effect on the olfactory learning, flight ability, and homing ability of *A*. *mellifera* [12]. Gao et al. [12] reported that mushroom bodies of *Tropilaelaps* mite-infested honey bees significantly increased when compared with uninfested honey bees, which may be related to a lower learning ability in the infested honey bees. In general, the mushroom body of insect brains is associated with antennal lobes containing primary olfactory neuropils [53]. As a result, the survival rate of the *Tropilaelaps*-infested honey bees can be much lower than that of uninfested honey bees [8,27]. In general, bees have two sets of wings that work together for flight performance [54] and make the air move and sound through the antennas to detect pheromones [54,55,56]. The result from our study showed that *Tropilaelaps* mites cause overt symptoms of wing deformities resulting in emerging honey bees that are unable to fly. Therefore, the colony was found to be less likely to survive as the number of infested honey bees increased. The degree of variations in the subsequent infestation among different body parts of the older and adult stages of honey bees could also be the cause and consequence of DWV infestation on either mites or bees in addition to the potential for variations in the preference of parasitic mites for different nutrient sources provided by different body parts of the bees. Unfortunately, we do not know whether variations in injury numbers among different body parts of *A*. *mellifera* were due to DWV or mite itself, or both. Nevertheless, our present findings provide a unique opportunity to investigate variations in the presence of mite feeding on different body parts across different developmental stages of honey bees.

## 5. Conclusions

In summary, the present study revealed that *T*. *mercedesae* mites infest throughout the entire development stage of *A*. *mellifera* honey bees. Specifically, the number of injuries was relatively high in the abdomens and the antennas. The presence of injuries and the decrease in infestation rates as the larvae progressed to the adult stage might be attributed to changes in the availability of nutrient contents, hygienic behaviors, and other unknown defense mechanisms against mite/virus infestations in the colony as the bees aged. This is the first study on the relationship either between *T*. *mercedesae* mite infestation rates and the injury numbers at different developmental stages or between the infestation rate and population size of honey bee *A*. *mellifera*. The advantage of this study is that it provides baseline information about the infestation rates of *T*. *mercedesae* mite in the larval and pupal stages of honey bees and the locations of injuries to both the pupal and adult stages of honey bees. It also provided important information about developing the population size of beehives when developing pest management programs for mites. Future studies could focus on how mechanisms that influence variation in the preference of *T*. *mercedesae* mites, coupled with other parasitic mites and viruses, among different body sizes of different bee species at different developmental stages and the abilities of the immune systems of each life stage of honey bees in response to such infestations to deepen our understanding of pest management for mites.

## Figures and Tables

**Table 1:** The relationship between the infestation rate and the number of injuries on larvae and pupae of *Apis mellifera*.

Developmental Stage of Honey Bee	Explanatory Fixed Variable	Estimate	SE	t-Value	*p*-Value
Larvae	Intercept	3.115	2.076	1.501	0.143
	Wound number	0.821	0.133	6.192	<0.001
Pupae	Intercept	0.325	0.380	0.855	0.399
	Wound number	0.117	0.024	4.822	<0.001

**Table 2:** Regression between *Tropilaelaps mercedesae* mite infestation rate and the population size of *Apis mellifera*.

Explanatory Fixed Variable	Estimate	SE	t-Value	*p*-Value
Intercept	62.62	8.37	7.477	<0.001
Population	−0.001	0.0003	−5.883	<0.001

## Data Availability

Not applicable.

## Supplementary Materials

The following supporting information can be downloaded at: https://www.mdpi.com/article/10.3390/ani13081318/s1, Figure S1. Feeding site of *Tropilaelaps mercedasae* on honey bee *Apis mellifera*. (A) Fresh integumental wounds (blue spots) and (B) scarred wounds (brown to blackspots) on prepupae. Figure S2. Photograph of feeding site caused by *Tropilaelaps mercedasae* on honey bee *Apis mellifera*. (A) antennas of an uninfested adult honey bee; (B) antennas of a crippled adult honey bee; (C) SEM picture of the antenna of a crippled adult honey bee; (D) the wing of the uninfested adult honey bee; (E) the wing of the crippled adult honey bee, (F) SEM picture of the wing of the crippled adult honey bee, (G) the abdomen of the uninfested tanned bodied pupa; (H) the abdomen of the infested tanned bodied pupa; and (I) SEM picture of the abdomen of the infested tanned bodied pupa.
