# Supplementary material for: Tropilaelaps mercedesae Infestation Is Correlated with Injury Numbers on the Brood and the Population Size of Honey Bee Apis mellifera
Source: Animals (Basel). 2023 Apr 12;13(8):1318. doi: 10.3390/ani13081318 (PMC10135255; doi:10.3390/ani13081318)
Supplement: Supplementary file 1 [file animals-13-01318-s001.zip › animals-2263550-supplementary.pdf]

animals-2263550 Supplementary **materials**

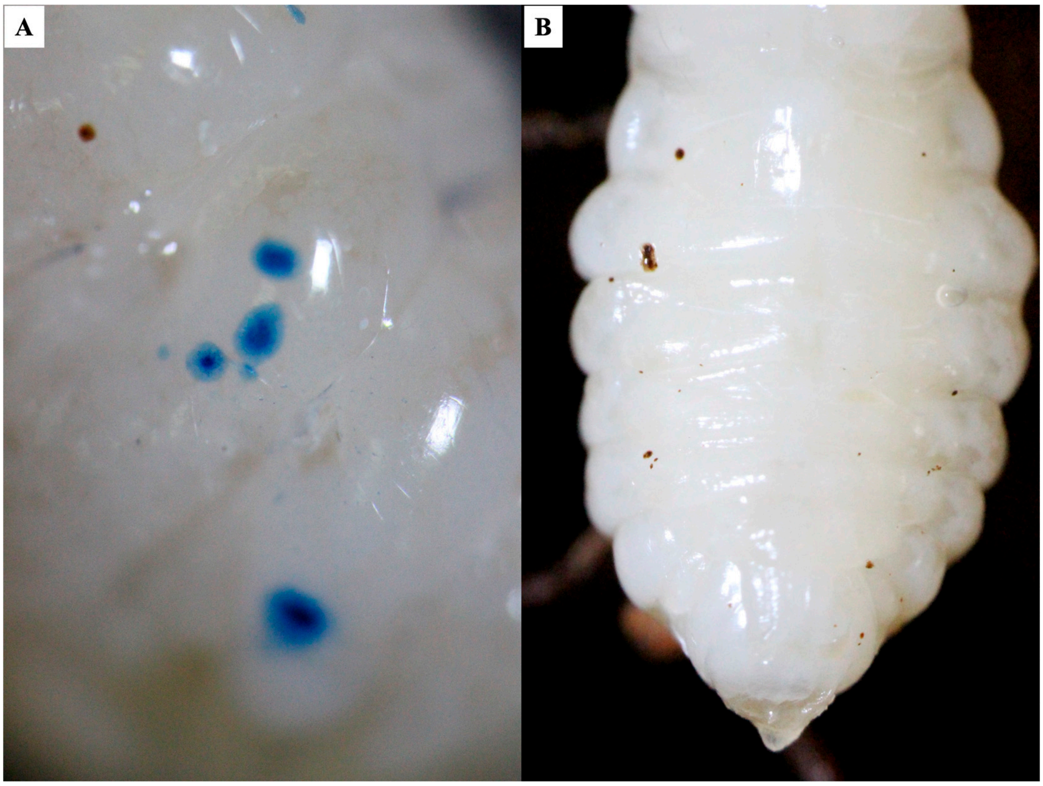

Figure S1. Feeding site of *Tropilaelaps mercedasae* on honey bee *Apis mellifera*. (A) Fresh integumental wounds (blue spots) and (B) scarred wounds (brown to blackspots) on prepupae.

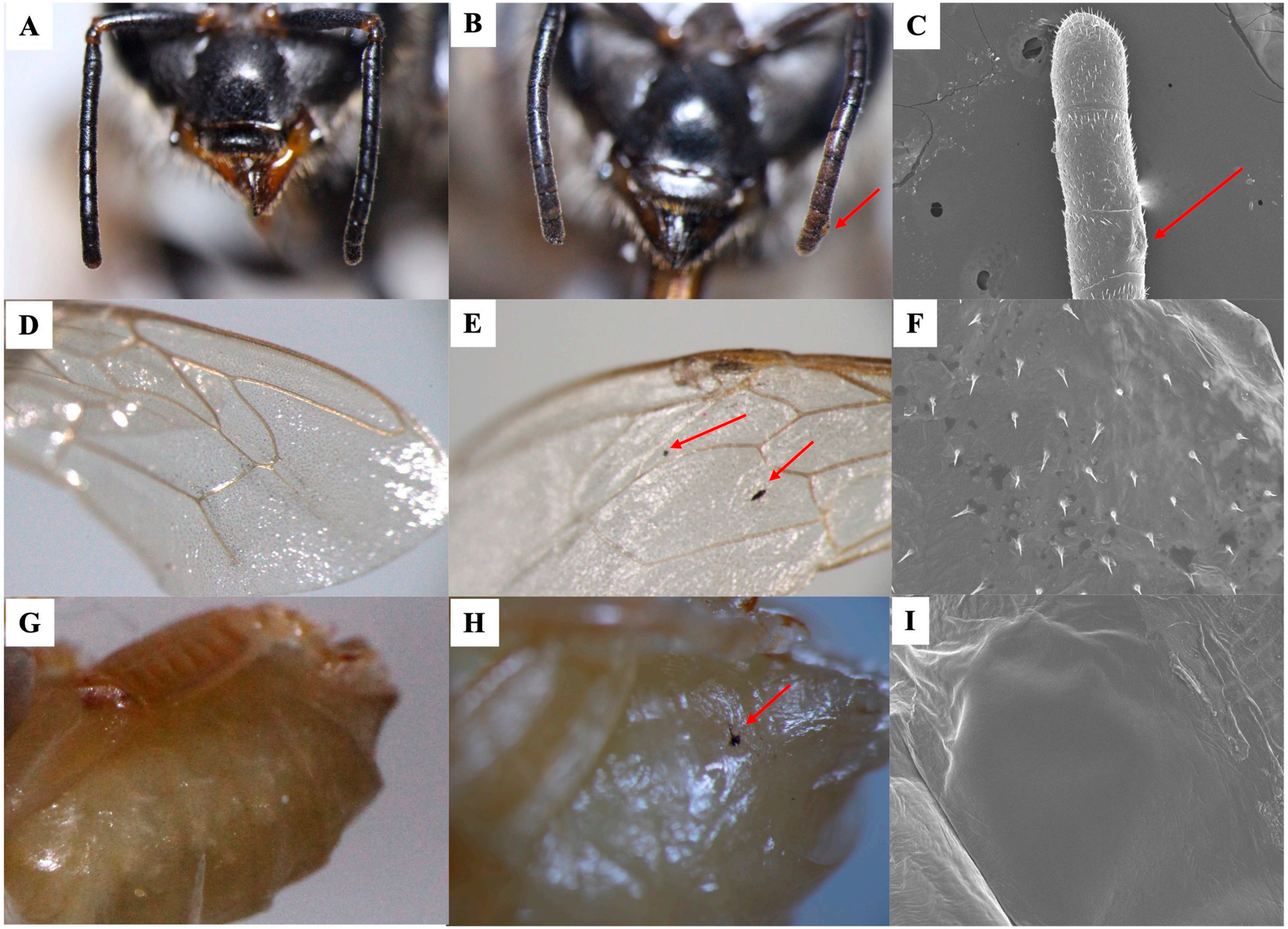

Figure S2. Photograph of feeding site caused by *Tropilaelaps mercedasae* on honey bee *Apis mellifera*. (**A**) antennas of an uninfested adult honey bee; (**B**) antennas of a crippled adult honey bee; (**C**) SEM picture of the antenna of a crippled adult honey bee; (**D**) the wing of the uninfested adult honey bee; (**E**) the wing of the crippled adult honey bee, (**F**) SEM picture of the wing of the crippled adult honey bee, (**G**) the abdomen of the uninfested tanned bodied pupa; (**H**) the abdomen of the infested tanned bodied pupa; and (**I**) SEM picture of the abdomen of the infested tanned bodied pupa.
